# Hematological Parameters Improve Prediction of Mortality and Secondary Adverse Events in Coronary Angiography Patients: A Longitudinal Cohort Study

**DOI:** 10.1097/MD.0000000000001992

**Published:** 2015-11-13

**Authors:** Crystel M. Gijsberts, Hester M. den Ruijter, Dominique P.V. de Kleijn, Albert Huisman, Maarten J. ten Berg, Richard H.A. van Wijk, Folkert W. Asselbergs, Michiel Voskuil, Gerard Pasterkamp, Wouter W. van Solinge, Imo E. Hoefer

**Affiliations:** From the Experimental Cardiology Laboratory, University Medical Center Utrecht (CMG, HMDR, DPVDK, GP, IEH); ICIN-Netherlands Heart Institute, Utrecht, The Netherlands (CMG, DPVDK); Department of Surgery, Yong Loo Lin School of Medicine, National University of Singapore (DPVDK); Cardiovascular Research Institute (CVRI), National University Heart Centre (NUHCS), National University Health System, Singapore, Singapore (DPVDK); Department of Clinical Chemistry and Hematology (AH, MJTB, RHAVW, WWVS, IEH); Department of Cardiology, University Medical Center Utrecht (FWA, MV); Durrer Center for Cardiogenetic Research, ICIN-Netherlands Heart Institute, Utrecht, The Netherlands (FWA); and Institute of Cardiovascular Science, Faculty of Population Health Sciences, University College London, London, UK (FWA).

## Abstract

Prediction of primary cardiovascular events has been thoroughly investigated since the landmark Framingham risk score was introduced. However, prediction of secondary events after initial events of coronary artery disease (CAD) poses a new challenge.

In a cohort of coronary angiography patients (n = 1760), we examined readily available hematological parameters from the UPOD (Utrecht Patient Oriented Database) and their addition to prediction of secondary cardiovascular events. Backward stepwise multivariable Cox regression analysis was used to test their ability to predict death and major adverse cardiovascular events (MACE). Continuous net reclassification improvement (cNRI) and integrated discrimination improvement (IDI) measures were calculated for the hematological parameters on top of traditional risk factors to assess prediction improvement.

Panels of 3 to 8 hematological parameters significantly improved prediction of death and adverse events. The IDIs ranged from 0.02 to 0.07 (all *P* < 0.001) among outcome measures and the cNRIs from 0.11 to 0.40 (*P* < 0.001 in 5 of 6 outcome measures). In the hematological panels red cell distribution width (RDW) appeared most often. The multivariable adjusted hazard ratio of RDW per 1 standard deviation (SD) increase for MACE was 1.19 [1.08–1.32], *P* < 0.001.

Routinely measured hematological parameters significantly improved prediction of mortality and adverse events in coronary angiography patients. Accurately indicating high-risk patients is of paramount importance in clinical decision-making.

## Abstract 2

Supplemental Digital Content is available in the text

## INTRODUCTION

Improvements in cardiovascular health care have significantly increased survival of coronary artery disease (CAD) patients.^1^ Consequently, the number of patients at risk for secondary events has risen. Despite being generally considered as high risk, this patient group is far from homogeneous; the risk of developing secondary adverse events varies from very low to very high.

The prediction of primary events has been studied for over half a century now, starting with the introduction of the landmark Framingham risk score.^2^ In addition to a clinical prediction model, many biomarkers have been evaluated for their ability to improve primary and secondary prediction, for example C-reactive protein,^3,4^ Cystatin C,^5,6^ and myeloperoxidase^7,8^ have shown to be associated with the risk of future events. Also, hematological parameters, mainly leukocyte-related parameters,^9,10^ have been reported to reflect the risk of primary cardiovascular events. More recently, high red blood cell distribution width (RDW), a measure of the variation of red blood cell size, has emerged as a predictor for atherosclerosis progression,^11^ CAD severity,^12^ and mortality.^13^

However, the secondary predictive value of traditional risk factors, for example, body mass index^14^ (BMI) and of biomarkers used in primary risk prediction is limited or remains unclear. Accurately predicting secondary risk is of paramount importance to the patient and their treating clinician in order to optimize secondary preventive measures for those at need. This may include novel and/or expensive therapies, for example, the proprotein convertase subtilisin/kexin (PCSK) type 9-inhibitors.^15^ To date, reliable tools that discriminate between high and low-risk patients with known CAD are lacking.

In the current study, we therefore sought to improve secondary risk prediction among coronary angiography patients. We did this by extending a clinical model containing risk factors, cardiovascular history, and angiographic characteristics with routinely measured and readily available hematological parameters. For this purpose, we used the Utrecht CORonary BIObank (UCORBIO) cohort^16^ in combination with hematological measurements from the Utrecht Patient-Oriented Database (UPOD)^17^ laboratory registrations.

## METHODS

### Study Population

We analyzed data from the UCORBIO cohort (clinicaltrials.gov identifier: NCT02304744), an observational cohort study of patients undergoing coronary angiography for any indication in the University Medical Center in Utrecht, The Netherlands. From October 2011 to February 2014, a total of 1904 patients were enrolled. For the current study, adult (>18 years) patients presenting with myocardial infarction (either ST-Segment Elevation Myocardial Infarction [STEMI] or Non-ST-Segment Elevation Myocardial Infarction [NSTEMI]), chest pain without release of cardiac enzymes (stable or unstable angina), dyspnea on exertion, silent ischemia, or screening for noncardiac surgery were selected (n = 1760). Patients with other indications for coronary angiography (coronary anomalies, screening for cardiac surgery, or heart transplant follow-up) were thus excluded (n = 144).

### Ethics, Consent, and Permissions

All patients provided written informed consent and the study conforms to the Declaration of Helsinki. The institutional review board of the University Medical Centre Utrecht approved of this study (reference number 11-183).

### Data Collection

The investigators completed standardized electronic case report forms at baseline based on the patient's medical files containing age, sex, cardiovascular risk factors, indication for angiography, medication use, angiographic findings, and eventual treatment of CAD. The definitions used for the baseline variables were published previously in more detail.^18^ The angiographic findings were categorized into 4 groups by the treating interventional cardiologist: no CAD, minor CAD (wall irregularities, <50% stenosis), single-vessel disease (1 vessel with >50% stenosis^19^), and multivessel disease (2 or 3 vessels with >50% stenosis).

### Hematological Parameters

The hematological parameters were obtained through complete blood count analysis at the moment of coronary angiography. The parameters that were used in this study comprised 56 routinely measured hematological parameters (listed in supplemental Figure 1, http://links.lww.com/MD/A515) from the UPOD database.^17^ A feature of the automated blood cell analyzer is that it not only reports the parameters requested by the physician, but all hematological parameters that it is capable of measuring. For example, when a physician requests a hemoglobin measurement, the analyzer also automatically determines the platelet count. Although this platelet count is not reported to the clinician, the analyzer stores the data. Periodically, all data captured within the blood cell analyzers are downloaded to a database format, and are cleaned and checked for integrity, making the data available for research.

The UPOD parameters contain information on red blood cell (RBC) numbers and characteristics, leukocyte numbers and characteristics, and platelet numbers and characteristics. All hematological parameters are measured using the Cell-Dyn Sapphire^20–22^ hematology analyzer (Abbott Diagnostics, Santa Clara, CA, USA). This analyzer is equipped with an integrated 488-nm blue diode laser and uses spectrophotometry, electrical impedance, laser light scattering (multiangle polarized scatter separation), and 3-color fluorescent technologies to measure morphological parameters of leukocytes, RBCs, and platelets for classification and enumeration. The morphological parameters entail the following 5 optical scatter signals for leukocytes: cell size (0° scatter, axial light loss), cell complexity and granularity (7° scatter, intermediate angle scatter (IAS)), nuclear lobularity (90° scatter, polarized side scatter (PSS)), depolarization (90° depolarized side scatter (DSS)), and viability (red fluorescence (FL-3), 630 ± 30 nm). For platelets, 2 optical scatter signals are measured: IAS scatter (7°, cell size) and PSS scatter (90°, granularity; internal structure). RBC parameters are measured or calculated on the basis of the impedance measurement. Reticulocytes are optically measured using IAS scatter (7°, cell size) and FL-1 fluorescence (RNA content). Throughout this paper, all values of hematological parameters are reported as multitudes of their standard deviation (SD) in order to ensure comparability of effect sizes among parameters with absolute values that vary strongly in their order of magnitude.

### Follow-Up

On a yearly basis, patients received a questionnaire to check for hospital admissions and occurrence of major adverse cardiovascular events (MACE). When the patient reported a hospital admission suspect for MACE or did not complete or return the questionnaire, the general practitioner or reported hospital was contacted for confirmation. In the case of hospitalization or death, medical records were obtained and the relevance of the event or the cause of death was determined. A panel of cardiologists adjudicated the occurrence of events. The composite end-point MACE was defined as any of the following clinical events: all-cause death, nonfatal myocardial infarction, unplanned revascularization; both cardiac (percutaneous coronary intervention (PCI) and coronary artery bypass grafting (CABG)) and noncardiac intervention, stroke, and admission for heart failure.

### Statistical Analysis

This study is reported in accordance with the STROBE guidelines for observational research.^23^ Baseline characteristics were reported as means and standard deviations for continuous variables and percentages for categorical variables, for the entire cohort and separately for patients who experienced MACE during follow-up and who did not.

First, we constructed a clinical risk prediction model. Covariates for this model were selected using a boosting technique for Cox regression models (R package “CoxBoost”^24^). The covariates considered were: age, sex, diabetes, hypertension, hypercholesterolemia, BMI, smoking, indication for angiography, angiographic CAD severity, treatment following angiography, history of PCI, history of CABG, history of acute coronary syndrome (ACS), history of cerebrovascular accident (CVA), history of peripheral arterial disease (PAD), kidney failure, use of ACE-inhibitor, use of beta-blocker, use of statin, use of P2Y12-inhibitor (clopidogrel, prasugrel, or ticagrelor), and use of diuretics. Age, sex, indication for angiography, angiographic severity of CAD, and treatment following angiography were considered mandatory covariates. The variables additionally selected using a boosting procedure were diabetes, history of PCI, history of ACS, history of PAD, kidney failure, and use of diuretics.

The coefficients of the clinical model parameters were refit for each outcome measure; the clinical model performed well for all outcome measures (AUCs ranging from 0.681 to 0.884).

For the identification of hematological parameters that could aid prediction of adverse events (total n = 56), first we evaluated mutual correlation of the parameters by means of a hierarchically clustered heatmap (supplemental Figure 1, http://links.lww.com/MD/A515). From each cluster of collinear parameters the parameter that showed the strongest relation with MACE was selected for the further analysis.

The remaining parameters (n = 37) were entered in 6 backward stepwise Cox regression models, 1 for each outcome measure: all-cause death, MACE, cardiovascular death, noncardiovascular death, re-PCI, and myocardial infarction.

From this procedure, the top 10 significant parameters for each outcome were added to the clinical parameters (which were forced to stay in the model, ie, mandatory covariates) and again backward stepwise Cox regressions were performed for the hematological parameters, rendering the final panels of hematological parameters for the 6 outcome measures while keeping the clinical parameters stable. In order to evaluate whether the predictive properties of hematological parameters differed across the indications for angiography and severities of CAD, we evaluated interaction terms.

For the panel of hematological parameters that appeared to be significantly related to adverse events and that were thus added to the clinical model, areas under the curve (AUCs) were compared to the clinical model alone using receiver operating characteristics (ROC) analysis. The R package “timeROC”^25^ was used for this purpose, which is based on the methods described by Chiang et al.^26^

Furthermore, according to the most recent epidemiological recommendations, continuous net reclassification improvement (cNRI) and integrated discrimination improvement (IDI) measures were calculated using the “survIDINRI” package^27,28^ in order to assess the improvement of risk prediction of adverse events. Continuous NRI was chosen over categorical NRI due to the lack of established meaningful risk categories in secondary risk prediction.^29^

Of the hematological parameters, RDW appeared to be performing particularly well. Therefore, baseline characteristics were additionally summarized by quartiles of RDW. All statistical analyses were performed using Rstudio^30^ and the R software package (version 3.1.2, Vienna, Austria).^31^ A *P* value of <0.05 was considered statistically significant. Missings were deleted listwise (<10%); no bias could be detected in terms of differing MACE occurrence between patients with and without missing covariates.

## RESULTS

### Patient Characteristics

The baseline results are presented for the entire cohort and stratified by the occurrence of MACE during follow-up (Table 1). On average, people with MACE were older (67.2 vs. 63.4, *P* < 0.001) than those without. Diabetes and hypertension were also more prevalent in the MACE group. The cardiovascular medical history of people with MACE more often showed ACS, PCI, CABG, CVA, and kidney failure. The indication for coronary angiography did not differ between the groups. The left ventricular ejection fraction (LVEF) was poorer and the angiographic burden of CAD was more severe in the MACE group. Consequently, the treatment was more invasive in the MACE group. The use of prasugrel, beta blockers, ACE inhibitors, statins, and diuretics was higher in the MACE group. During a median follow-up time of 779 days, 99 deaths and 368 MACEs occurred.

**TABLE 1:**
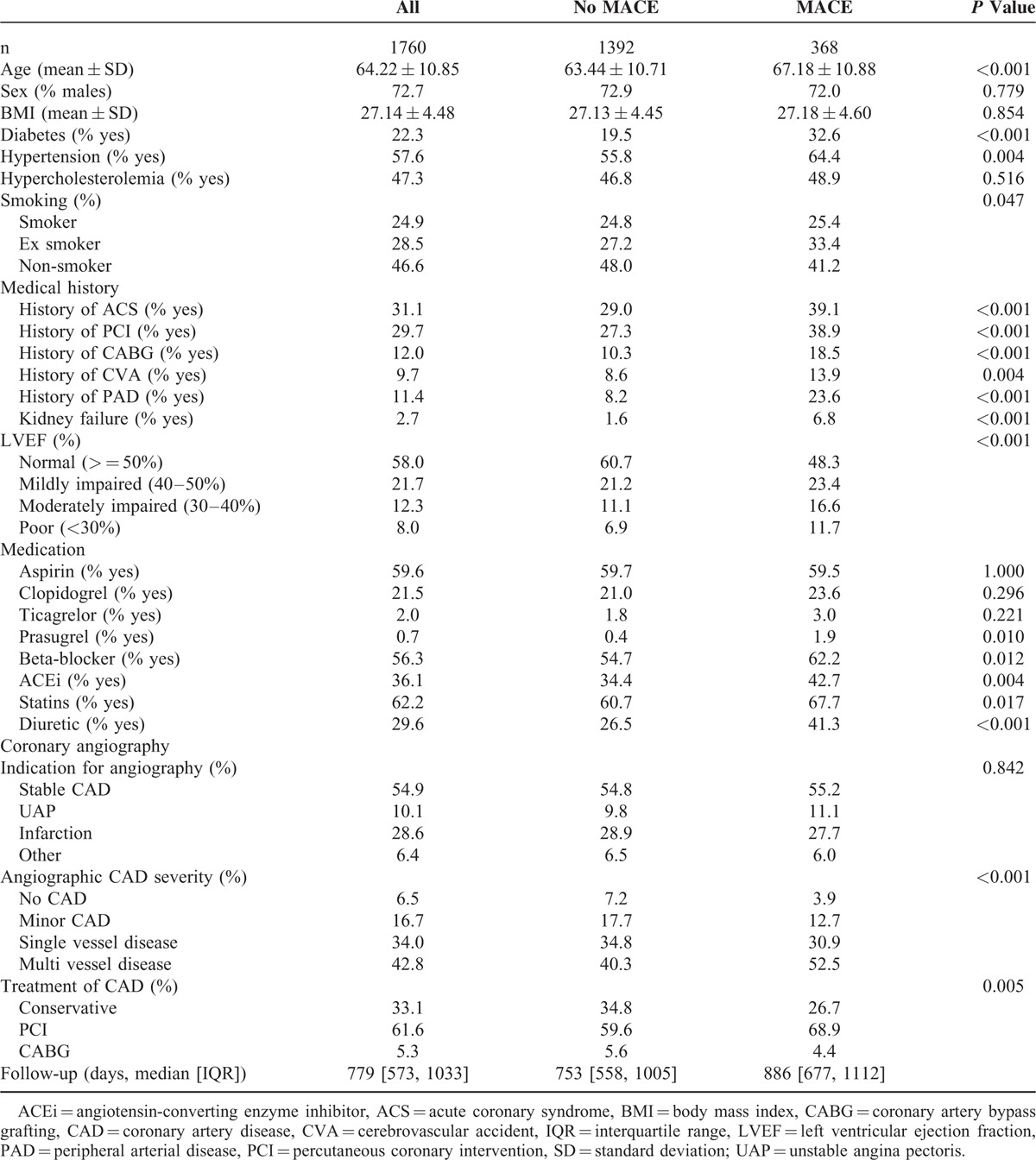
Baseline Characteristics of UCORBIO Patients, Stratified by MACE During Follow-Up

### Hematological Parameters

In Table 2, the baseline levels of the hematological parameters of interest (n = 37) are displayed by the occurrence of MACE during follow-up. Sixteen parameters differed significantly between patients with and without MACE during follow-up: leukocyte count, monocyte count, eosinophil count, basophil count, lymphocyte %, hemoglobin, % RCBs larger than 120fL, RDW, mean corpuscular hemoglobin concentration (MCHC), mean platelet volume (MPV), mean neutrophil cell size, mean neutrophil granularity/lobularity, mean neutrophil red fluorescence, lymphocyte cell size coefficient of variation (CV), platelet granularity CV, and reticulocyte hemoglobin concentration (CHCr).

**TABLE 2:**
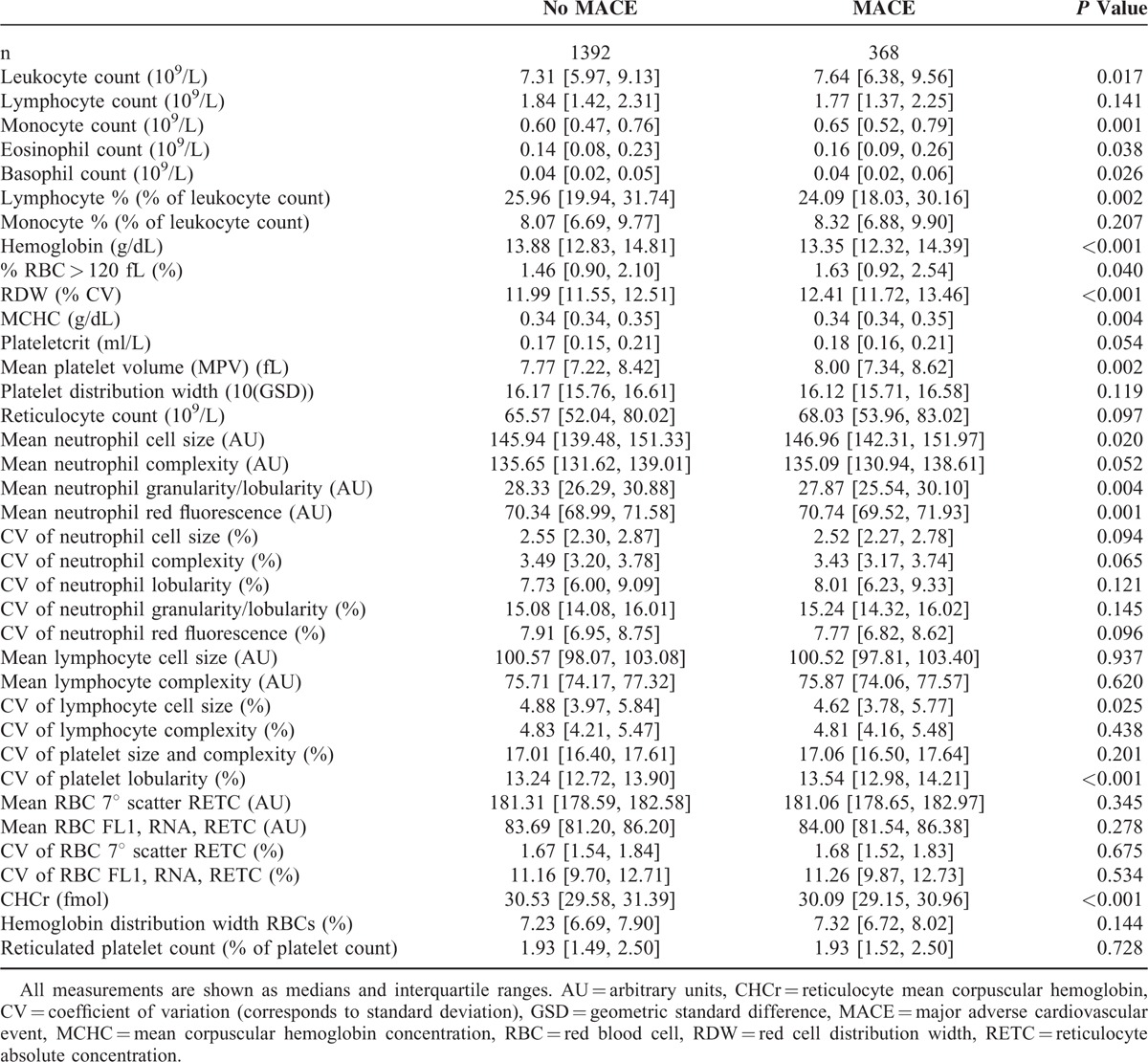
Baseline Values of Hematological Parameters, Stratified by the Occurrence of MACE During Follow-Up

### Risk Prediction With Hematological Parameters

For each outcome, the 10 best predictive hematological parameters (derived from backward stepwise analysis as described in the Methods section) were added to the clinical model containing age, sex, diabetes, indication for angiography, angiographic CAD severity, history of PCI, history of ACS, history of PAD, kidney failure, treatment following angiography (conservative, PCI or CABG), and use of diuretics. The hematological parameters that remained significantly associated with the outcome of interest are displayed in Table 3. Panels of hematological parameters, sized between 3 and 8 parameters, were significantly predictive on the top of the clinical model. For all outcomes except re-PCI (for which the panel only contained leukocyte parameters) the panels consisted of parameters from both leukocyte and RBC origin. In particular, RDW was abundant and appeared in 4 panels (for MACE, all-cause death, noncardiovascular death, and myocardial infarction), thus showing broadly applicable predictive properties.

**TABLE 3:**
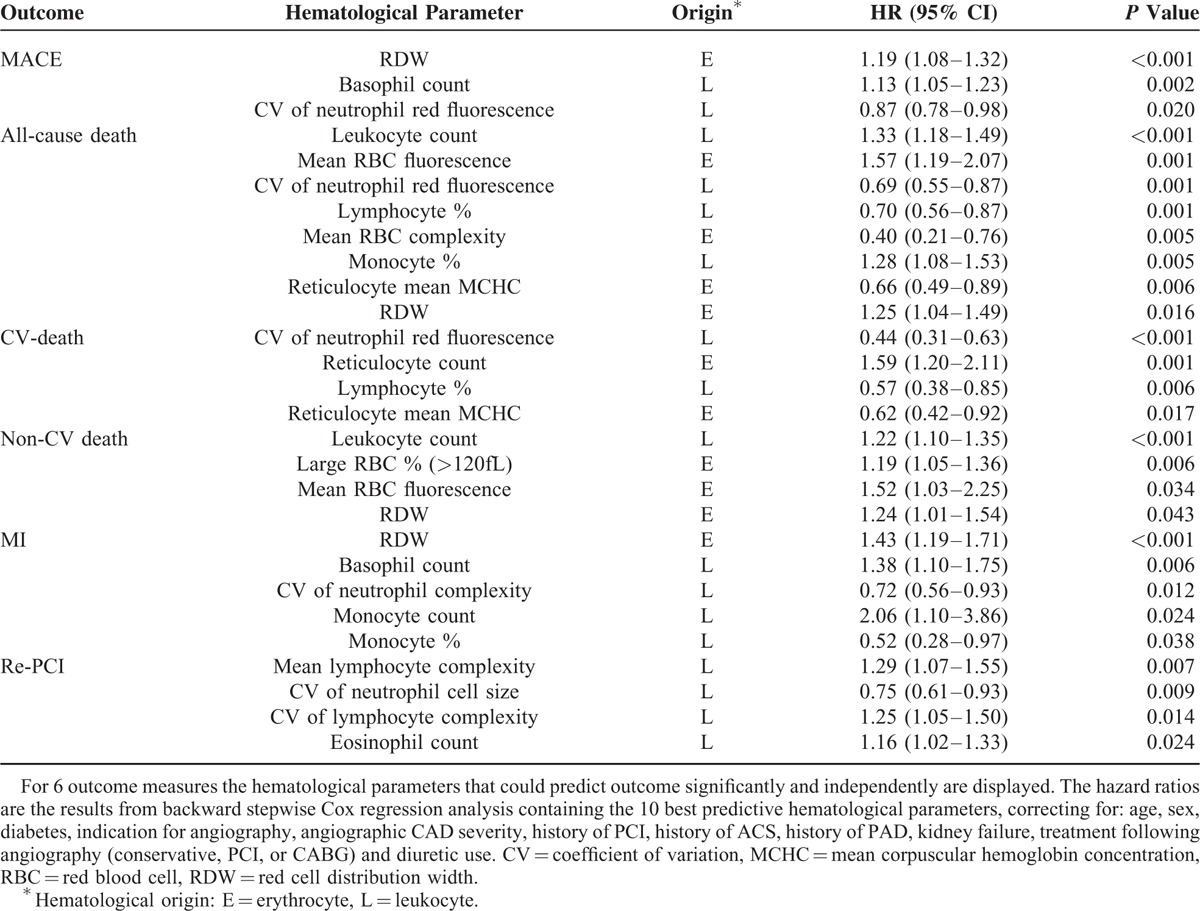
Multivariable Adjusted Hazard Ratios Derived From Backward Stepwise Cox Regression

The predictive properties of the hematological panels did not differ by indication for angiography or by severity of CAD for any of the outcome measures, as reflected by nonsignificant interaction terms.

### Improvement of Risk Prediction

**FIGURE 1:**
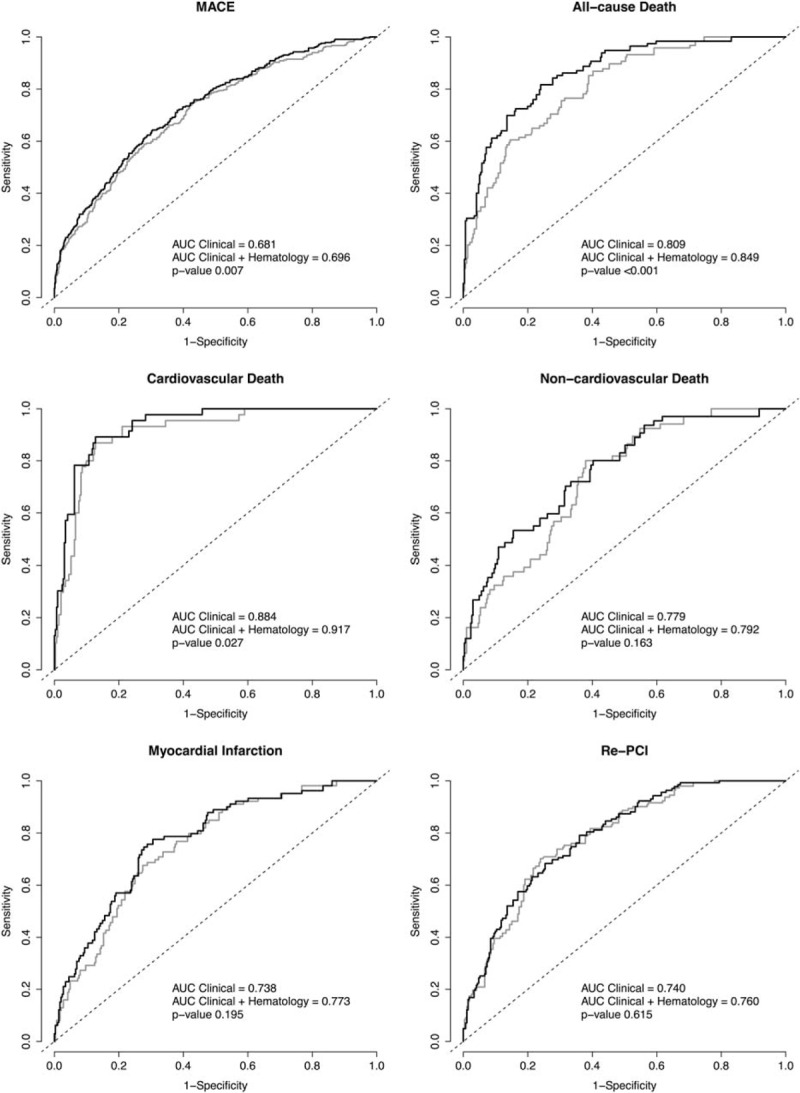
. ROC curves of models for a clinical model with and without hematological parameters *P* values are given for difference between the area under the curve (AUC) of the clinical model plus hematological parameters (black line) as compared with the clinical model only (gray line). The hematological parameters added to the model are as stated in Table 3.

Measures of prediction improvement were calculated for the prediction models extended with hematological parameters as compared with the basic clinical model. The cNRIs and IDIs resulting from this comparison are presented in Table 4. Additionally, Figure 1 shows the result from traditional ROC analysis for MACE, all-cause death, cardiovascular death, noncardiovascular death, myocardial infarction, and re-PCI. Supplemental Figures 2 and 3, http://links.lww.com/MD/A515, provide visual representations of the changes in predicted risk after addition of hematological parameters. For MACE, the IDI—indicating the change in the difference of the predicted risk between patients with events and patients without events in the model extended with hematological parameters as compared with the model without hematological parameters^32^—was the cNRI for MACE—indicating the proportion of individuals that were justly reclassified into a higher or lower risk by the extended model^33^—was 0.17 (95% CI: 0.08–0.23, *P* < 0.001). Additionally, for all-cause death, cardiovascular death, noncardiovascular death, myocardial infarction and re-PCI significant, and substantial IDIs and cNRIs (except for noncardiovascular death, *P* = 0.059) were calculated, thus demonstrating to provide improvement of prediction for a diversity of adverse outcomes.

**TABLE 4:**
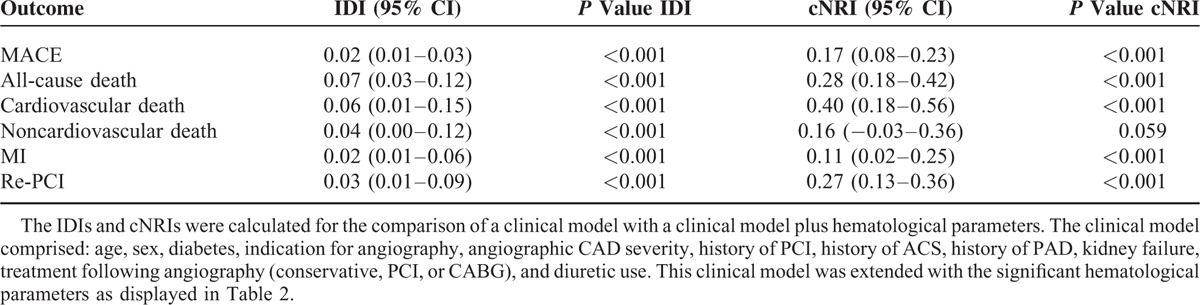
Measures of Prediction Improvement (IDIs and cNRIs) Upon Addition of Hematological Parameters

### Association of Patient Characteristics With RDW

**FIGURE 2:**
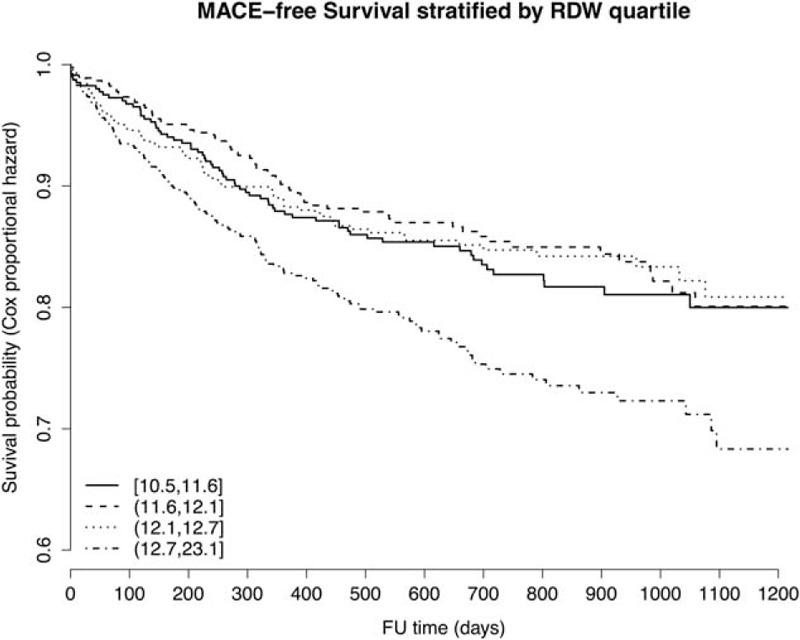
Multivariable adjusted MACE-free survival by RDW quartiles MACE-free survival plot by RDW quartiles. The results are derived from Cox regression analysis adjusting for age, sex, diabetes, smoking, indication for coronary angiography, angiographic severity of CAD, history of PCI, history of ACS, history of PAD, kidney failure, treatment of CAD, and diuretic use.

RDW was predictive of 4 of 6 outcome measures. In order to better understand the patient groups in which this parameter is elevated we evaluated baseline patient characteristics by quartiles of RDW (supplemental Table 1, http://links.lww.com/MD/A515). We found that RDW was positively associated with age, BMI, diabetes, and hypertension prevalence, a history of CABG, PAD, kidney failure, use of beta-blocker, and diuretics. RDW was negatively associated with LVEF. Multivariable adjusted survival by RDW quartile is depicted in Figure 2.

## DISCUSSION

In this study, we showed that the addition of readily available hematological parameters to a clinical model could significantly improve prediction of death and adverse events in coronary angiography patients. Efforts should be pursued to translate our findings into a clinically applicable risk score. More accurate identification of high-risk patients can lead to improved follow-up of patients at highest risk and treatment of those who will benefit most, thereby lowering the burden of cardiovascular morbidity and mortality.

### Predictive Properties of Hematological Parameters

Among the hematological parameters tested in our study for their predictive value, RDW was most abundant. The RDW is routinely measured by dividing the SD of the mean corpuscular volume (MCV) distribution by the mean of the MCV and then multiplying it by 100 to provide a percentage.^34^ High RDW thus reflects a higher variation in RBC volumes, also referred to as anisocytosis. Traditionally, RDW is measured to aid differential diagnosis of anemias. However, ours and other studies have shown that higher RDW is associated with poorer outcome for traumatic injuries,^35^ sepsis,^36–38^ stroke,^39,40^ myocardial infarction,^12,41–43^ PCI,^44–46^ heart failure,^47–51^ and in the general population.^13^ In the current study, we confirmed that RDW independently or in combination with other hematological parameters predicts mortality and secondary adverse events in a coronary angiography population. In addition to prior studies, we demonstrated that the addition of hematological parameters to clinical data can indeed improve risk prediction using modern statistical techniques (IDI^32^ and cNRI^33^).

In addition to RDW, we found predictive potential for several leukocyte parameters; the CV of neutrophil red fluorescence (for MACE, all-cause death and MI), basophil counts (MACE and MI), lymphocyte % (for all-cause death and CV-death), monocyte % (for all-cause death and MI), mean RBC red fluorescence (for all-cause death and non-CV death), and leukocyte count (for all-cause death and non-CV death). Some of these parameters, leukocyte, monocyte, and lymphocyte counts, have been described before,^9,52^ but the predictive values of the CV of neutrophil red fluorescence and basophil counts are largely uncovered in the current literature. To our knowledge, the CV of neutrophil red fluorescence has not been mentioned in the context of cardiovascular disease before. However, in a patient group with symptomatic PAD, basophil count was not an independent predictor of MACE^53^ and also among community-dwelling elderly, basophil count was not significantly associated with a history of cardiovascular disease (odds ratio 1.21 [0.98–1.50]).^54^ Possibly, these populations were too homogeneous for basophil counts to offer additive discriminative value. One can imagine that within the general population basophil counts are low, with little variation. The same could apply to a very sick population (like symptomatic PAD patients^53^), who would have high basophil counts with little variation. In our study population, patients with angiographic CAD severity ranging from no CAD or minor CAD to triple vessel disease are enrolled, thus representing a relatively heterogeneous population.

### Red Blood Cells and Cardiovascular Disease

Several mechanisms relate cardiovascular disease to changes in RBC characteristics. First, atherosclerosis is hallmarked by oxidative stress. Upon oxidative stress, RBCs adopt a more irregular and heterogeneous conformation.^55^ RBCs can encounter oxidative stress by passing through jeopardized tissues or microenvironments, such as atherosclerotic plaques.^56^ The oxidative changes can cause an increase in RBC degradation and turnover, resulting in a higher proportion of small RBCs and thus increased anisocytosis (higher RDW).

Second, inflammation is a keystone of atherosclerosis and several proinflammatory cytokines (eg, IL-6^57^) have been related to increased RDW. Inflammatory cytokines such as interferon-γ and tumor necrosis factor, which are elevated in CAD,^58^ suppress erythropoiesis and stimulate phagocytosis of senescent RBCs, thereby increasing anisocytosis.^59^

Third, CAD is frequently accompanied by some degree of kidney function impairment.^60^ Erythropoietin (EPO) is a hormone produced in the renal cortex promoting erythropoiesis and erythrocyte maturation. Disturbances in EPO production^34^ and responsiveness^61^ have been related to increased RDW. As EPO levels decrease upon inflammation,^62^ a disturbed erythropoiesis and thereby an increase in RDW can be observed.^63^

### Secondary Risk Prediction Improvement in Clinical Practice

In the current study, we showed that risk estimation following coronary angiography can be significantly improved by addition of hematological parameters. These parameters are readily available in the vast majority of medical centers as they are measured with every differential blood count on automated hematology analyzers. Clinical risk prediction rules therefore might be effortlessly extended with a panel of hematological parameters, resulting in more accurate identification of high-risk individuals. These high-risk individuals need to be identified in order to justly provide expensive secondary prevention therapies with limited availability, such as the soon-to-come PCSK9 inhibitors.

While our results are promising, external validation is warranted in order to establish the clinical usefulness of hematological parameters in the context of risk prediction.

### Limitations

In our cohort lipid levels were not available. Therefore, established secondary risk prediction scores as the PROCAM, Framingham, SCORE, or SMART-score^64^ could not be applied.

Also, the duration of symptoms and the delay between acute onset of chest pain and the moment of coronary angiography might affect the levels of hematological parameters. However, the majority of our cohort consists of stable CAD patients (55%) without acute symptoms. Hence, such effects on RDW are very unlikely. In patients with >1-day delay between symptom onset and angiography, we investigated the possible correlation between the delay and RDW, which yielded no significant result (*P* = 0.399). A study conducting repetitive measures would be necessary for evaluating changes of hematological parameters throughout the course of CAD, such as the BioMarcs^65^ program.

In the current analyses, we did not consider interaction terms for, for example, sex. It is possible in the light of differing reference values for hematological parameters that different coefficients need to be applied to men and women. Future research has to evaluate the need for sex interaction terms in a clinically applicable risk prediction model.

## CONCLUSIONS

Hematological parameters, particularly the RDW, can significantly improve the prediction of secondary adverse events in a coronary angiography population. This will help identify high-risk patients more accurately and tailor secondary prevention based on individual risk. The clinical potential of a risk score extended with hematological parameters needs to be evaluated further.
